# A systematic review of clinical outcomes on the WHO Category II retreatment regimen for tuberculosis

**DOI:** 10.5588/ijtld.17.0705

**Published:** 2018-10-01

**Authors:** D. B. Cohen, J. Meghji, S. B. Squire

**Affiliations:** * Liverpool School of Tropical Medicine, Liverpool, UK; † Malawi-Liverpool-Wellcome Clinical Research Programme, Blantyre, Malawi; ‡ University of Sheffield, Sheffield, UK

**Keywords:** relapse, recurrent, streptomycin, cure, isoniazid

## Abstract

**OBJECTIVE:**

To assess the clinical outcomes of patients prescribed the World Health Organization (WHO) Category II retreatment regimen for tuberculosis (TB).

**DESIGN:**

A systematic review of the literature was performed by searching Medscape, Embase and Scopus databases for cohort studies and clinical trials reporting outcomes in adult patients on the Category II retreatment regimen.

**RESULTS:**

The proportion of patients successfully completing the retreatment regimen varied from 27% to 92% in the 39 studies included in this review. In only 2/39 (5%) studies was the treatment success rate > 85%. There are very few data concerning outcomes in patients categorised as ‘other’, and outcomes in this subgroup are variable. Of the five studies reporting disaggregated outcomes in human immunodeficiency virus (HIV) positive people, four demonstrated worse outcomes than in HIV-negative people on the retreatment regimen. Only four studies reported disaggregated outcomes in patients with isoniazid (INH) resistance, and treatment success rates varied from 11% to 78%.

**CONCLUSION:**

Clinical outcomes on the Category II retreatment regimen are poor across various populations. Improvements in management should consider the holistic treatment of comorbidity and comprehensive approaches to drug resistance in patients with recurrent TB, including a standardised approach for the management of INH resistance in patients who develop recurrent TB in settings without reliable access to comprehensive drug susceptibility testing.

## Abstract (FR)

**OBJECTIF:**

Evaluer les résultats cliniques des patients auxquels on a prescrit pour la tuberculose (TB) le protocole de retraitement de l'Organisation Mondiale de la Santé (OMS) de Catégorie II.

**SCHÉMA:**

Une revue systématique de la littérature a été réalisée grâce à une recherche sur les bases de données Medscape, Embase et Scopus sur les études de cohorte ou les essais cliniques rapportant les résultats de patients adultes sous protocole de retraitement de Catégorie II.

**RÉSULTATS:**

La proportion de patients qui ont achevé avec succès le protocole de retraitement a varié de 27% à 92% dans les 39 études incluses dans la revue. Le taux de succès du traitement n'a été de > 85% que dans 2/39 (5%)études. Il y a peu de données relatives aux résultats des patients classés comme « autres » et les résultats de ce sous-groupe sont variables. Sur les cinq études rapportant des résultats désagrégés chez les personnes positives à l'infection par le virus de l'immunodéficience humaine (VIH), quatre ont démontré des résultats plus médiocres que chez les personnes VIH négatives sous protocole de retraitement. Seules quatre études ont rapporté des résultats désagrégés chez des patients ayant une résistance à l'isoniazide (INH) et le taux de succès du traitement a varié de 11%à 78%.

**CONCLUSION:**

Les résultats cliniques du protocole de retraitement de l'OMS de Catégorie II sont médiocres dans toutes sortes de populations. Les améliorations de la prise en charge devraient porter sur un traitement holistique des comorbidités et des approches intégrées de la pharmacorésistance des patients atteints de TB récurrente, incluant une approche standardisée de la prise en charge de la résistance à l'INH chez les patients qui développent une TB récurrente dans des contextes sans accès fiable à de tests exhaustifs de pharmacosensibilité.

## Abstract (SP)

**OBJETIVO:**

Evaluar los desenlaces clínicos de los pacientes que han recibido un esquema de retratamiento de la tuberculosis (TB) de la Categoría II de la Organización Mundial de la Salud (OMS).

**MÉTODO:**

Se llevó a cabo una revisión sistemática de las publicaciones científicas tras la búsqueda en las bases de datos Medscape, Embase y Scopus de estudios de cohortes o ensayos clínicos que comunicaban los desenlaces de pacientes adultos tratados con un esquema de retratamiento de la TB de la Categoría II.

**RESULTADOS:**

La proporción de pacientes que completaron con éxito el esquema de retratamiento osciló entre 27% y 92% en los 39 estudios incluidos en la revisión. Solo en dos de los 39 estudios (5%) la tasa de éxito terapéutico fue > 85%. Se encontraron pocos datos sobre los desenlaces de pacientes en la categoría ‘otros’ y en este subgrupo los resultados fueron variables. De los cinco estudios que presentaban desenlaces desglosados en función de la infección por el virus de la inmunodeficiencia humana (VIH), cuatro revelaban desenlaces más desfavorables con el esquema de retratamiento en las personas positivas que en las personas no afectadas por el virus. Solo cuatro estudios presentaban desenlaces desglosados en los pacientes con resistencia a isoniazida (INH) y las tasas de éxito terapéutico oscilaban entre 11% y 78%.

**CONCLUSIÓN:**

Los desenlaces clínicos alcanzados con un esquema de retratamiento de la Categoría II de la OMS son deficientes en una diversidad de poblaciones. Los progresos en la gestión de los casos deben tener en cuenta el tratamiento holístico de las enfermedades concomitantes y comportar estrategias integrales frente a la farmacorresistencia en los pacientes con TB recurrente, que incluyan un esquema normalizado de tratamiento de la resistencia a INH en las personas con recurrencia de la TB, en los entornos que no cuentan con un acceso fiable a pruebas completas de sensibilidad a los medicamentos.

OF THE 6.6 MILLION PEOPLE worldwide reported to have tuberculosis (TB) in 2016, approximately 300 000 had previously received treatment.^1^ Until recently, in settings with low or medium prevalence of multidrug-resistant TB (MDR-TB, defined as TB resistant to at least isoniazid [INH, H] and rifampicin [RMP, R]) and no routine access to drug susceptibility testing (DST), the World Health Organization (WHO) recommended that previously treated patients with TB be prescribed an empirical regimen comprising 2 months of RMP, INH, pyrazinamide (Z), ethambutol (E) and streptomycin (SM, S), followed by 1 month of RHZE and 5 months of RHE.^2^ This ‘Category II’ regimen was devised based on expert opinion; however, there have been no clinical trial data on which to assess its efficacy. WHO guidelines published in 2017 recommended that the Category II regimen should no longer be prescribed and that DST be conducted to inform the choice of treatment regimen.^3^ However, given the lack of data on which to base decisions regarding retreatment, this recommendation is presented only as a good practice statement.

In 1991, the World Health Assembly set a target to cure 85% of patients who receive treatment for sputum smear-positive TB.^4^ In recent years, giant steps have been taken to meet these targets, with 83% of new cases now successfully cured.^1^ However, rates of successful treatment are consistently lower in patients prescribed the retreatment regimen. A meta-analysis of studies evaluating outcomes in patients who were retreated for TB identified only six cohort studies reporting outcomes for the Category II regimen.^5^ These studies demonstrated treatment failure rates of between 0% and 27% in patients infected with a fully susceptible strain of TB; however, in patients with INH monoresistance (INH-R) and either mixed or unknown resistance patterns, failure rates were even higher, at respectively 18–44% and 9–45%. This meta-analysis only included patients with microbiologically confirmed TB, and therefore excluded a large number of patients who had sputum smear-negative or extra-pulmonary TB (EPTB), and in whom outcomes are likely to be even worse.^6^

Patients receiving Category II retreatment are classified into four categories: sputum smear-positive relapse, treatment after failure, treatment after default (now known as loss to follow-up), and ‘other,’ which consists largely of patients who have smear-negative or extra-pulmonary recurrence. The underlying disease processes, and therefore clinical outcomes, may vary significantly between these categories. For example, those with true relapse almost certainly have TB and are therefore likely to respond to retreatment, whereas those classified as ‘other’ may be presenting with symptoms caused by an alternative disease (e.g., cancer or bronchiectasis), for which anti-tuberculosis treatment will be ineffective. In addition, although drug resistance is more prevalent in people with recurrent TB than people presenting with a first episode of TB, only a minority are infected with a resistant strain in most settings. Moreover, although recent WHO guidelines advocate phasing out the Category II regimen in favour of treatment based on DST results, a large number of patients with recurrent disease will not have micro-biologically confirmed disease; decisions will therefore have to be made without information about resistance patterns on which to base treatment regimens.

We performed a protocol-driven systematic review of the literature^7^ to examine clinical outcomes on a TB retreatment regimen in both microbiologically confirmed and unconfirmed cases, including those registered as ‘other.’ We examined outcomes in different groups of patients to consider possible factors that may be associated with the poor outcomes seen in patients with recurrent TB.

## METHODOLOGY

### Information sources and search strategy

Medscape, Embase and Scopus databases were searched using the following search terms: ‘Tuberculosis’ ‘retreat^*^’, (Category II) and (Category 2). Searches were limited to manuscripts published in English after 1991 and concerning human subjects. A manual search of the journal *Public Health Action* was performed, as it was a relatively new journal and likely to publish relevant manuscripts. Reference lists of identified studies were examined for further relevant publications.

### Eligibility

Prospective or retrospective cohort studies or clinical trials reporting outcomes in adult patients prescribed the Category II TB retreatment regimen were included. The review included studies published after 1991, as this was when the standardised retreatment regimen was introduced. Only studies published in English were included. Studies reporting on patients receiving regimens for TB other than the standard WHO Category II regimen, studies that did not use standard WHO definitions for reporting TB outcomes (cured, completed, failed, died, lost to follow-up, not evaluated^8^), and reports including only patients with MDR-TB receiving retreatment regimen were excluded.

### Study selection and data collection process

After removal of duplicates, study titles, then abstracts, and finally full texts of manuscripts were reviewed. Two reviewers (DBC and JM) assessed manuscripts independently at each stage and, if discrepancies arose, consensus was reached by discussion between the two reviewers and a third reviewer (SBS).

Data were extracted and entered directly into a standardised spreadsheet and included details of the study design, setting, population characteristics, treatment regimens, reporting definitions, number of study participants and baseline smear status. Outcomes for the total study population and subgroups, including TB category, HIV status and INH susceptibility pattern, were recorded. An evaluation of the generalisability of each study was made based on inclusion criteria and data source.

### Summary measures and synthesis of results

The specific endpoint of interest was the proportion of patients in whom an outcome of ‘treatment success’ was recorded. Treatment success was a composite of ‘cured’ and ‘completed’. ‘Cured’ was defined as being smear-negative at the end of treatment having started smear-positive. ‘Completed’ was defined as finishing a course of treatment, but not meeting the criteria for cure.^9^ The proportion of patients with a successful treatment outcome was then calculated for each study. Data were analysed using STATA v12 (StataCorp, College Station, TX, USA) and *I*^2^ estimates derived for the assessment of heterogeneity between studies.

## RESULTS

**Figure 1:**
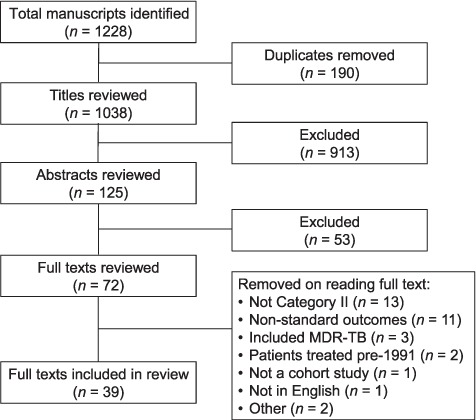
Study selection. MDR-TB = multidrug-resistant tuberculosis.

A total of 1038 publications were identified after removing duplicates (Figure 1). After examination of 72 full-text articles, 39 studies were included in the final analysis (Table 1).^10–48^ The majority of the studies included in the review (33/39) were retrospective cohorts based on analyses of routinely collected data in local or national TB registers. The majority of the studies were conducted in Asia (predominantly India) or Africa. See Appendix Table A^*^ for study details.

**Table 1:**
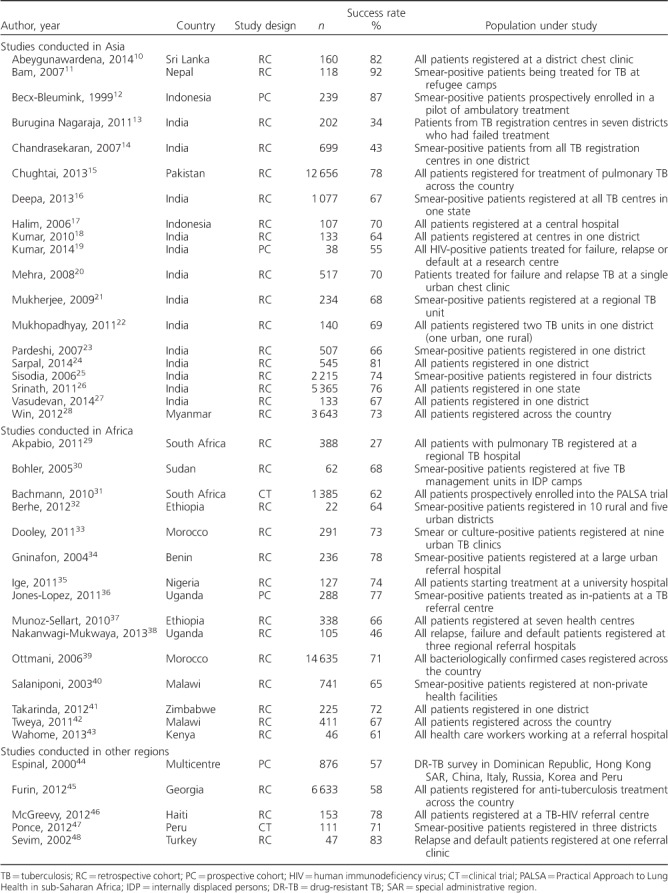
Studies describing outcomes on TB retreatment regimen

### Overall outcomes on tuberculosis retreatment regimen

The proportion of patients successfully completing a retreatment regimen ranged from 27% to 92%. There was significant heterogeneity between studies (*I*^2^ = 0.95), which precluded calculation of a pooled estimate. Only 2/39 (5%) studies met the target of 85%, successful treatment. The treatment success rate was <75% in 29 (74%) studies, and <50% in four studies.

### Outcomes on tuberculosis retreatment regimen by category

**Figure 2:**
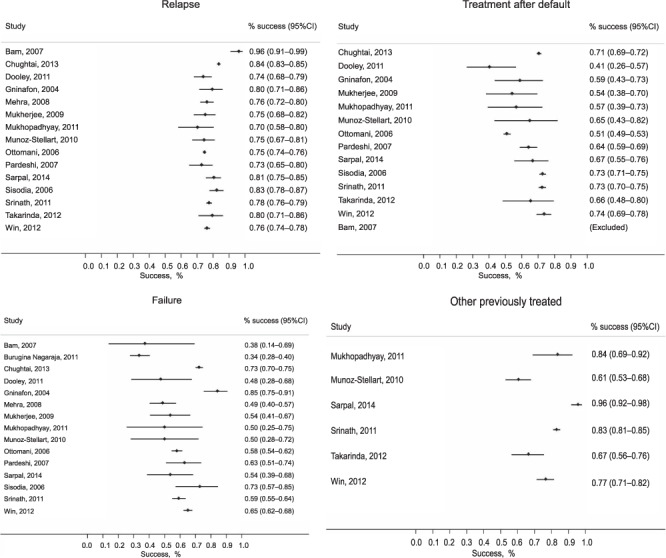
Treatment outcomes according to TB retreatment category: A) retreatment after relapse; B) retreatment after default (loss to follow-up); C) treatment after failure; and D) other. TB = tuberculosis.

The successful completion rate in patients with TB relapse was ⩾70% in all studies, and confidence intervals (CIs) around these estimates tended to be small (Figure 2A). Outcomes in patients registered as having returned to treatment after default or having failed treatment varied more widely, at 41–74% and 34–85%, respectively (Figures 2B and 2C). Very few studies specifically reported outcomes in patients registered as ‘other’. The rate of successful treatment in this group varied from 61% to 84% (Figure 2D).

### Outcomes on a tuberculosis retreatment regimen by isoniazid resistance

Disaggregated data for outcomes among patients with confirmed INH resistance were reported in four studies, involving only 217 patients (Table 2). Three of the four studies were conducted in India, and in these the prevalence of INH-R ranged from 12% to 28%. The largest of the studies aimed to compare outcomes in patients with and without INH-R, and found that 44% of those with INH-R had poor treatment outcomes, compared with 31% of those infected with a susceptible strain (adjusted relative risk [aRR] 1.46, 95%CI 1.19–1.78).^16^

**Table 2:**
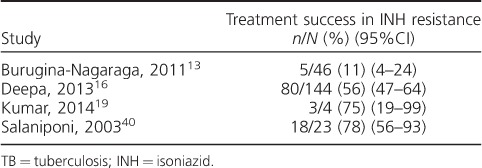
TB retreatment outcomes by INH resistance status

### Outcomes on tuberculosis retreatment regimen by human immunodeficiency virus status

Only five studies compared outcomes in HIV-positive and HIV-negative patients (Table 3). Two were conducted in African countries with generalised HIV epidemics (Uganda and Zimbabwe), one in Haiti (HIV prevalence < 2%), one in Indonesia (HIV prevalence 0.5%), and one in India (HIV prevalence < 0.5%). In 4/5 studies, outcomes were worse in HIV-infected patients. In the one study that reported higher success rates in HIV-positive people, the number of HIV-negative patients was very small. Jonez-Lopez et al. reported an adjusted odds ratio (OR) of 2.16 (95%CI 1.01–4.61) for unsuccessful outcome in HIV-positive patients in Uganda, with factors associated with death being CD4 count < 50 cells/ml, no antiretroviral therapy (ART) and Karnofsky score < 70%.^36^ In a study by McGreevy et al., follow-up data demonstrated that only 46% of HIV-positive patients remained alive, in ART care and TB-free after a median of 36 months of follow-up.^46^

**Table 3:**
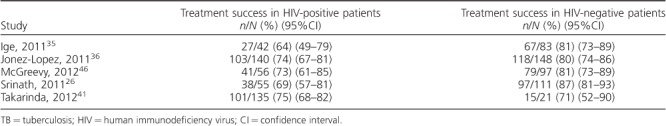
TB retreatment outcomes by HIV status

## DISCUSSION

This review demonstrated that, in keeping with previous reports, rates of successful treatment using the WHO Category II regimen for TB retreatment were consistently lower than the target of 85%. We presented disaggregated data available for TB retreatment subgroups; although data were limited, outcomes may have been worse in HIV-infected adults, those with INH-R, and patients with smear-negative TB and EPTB registered as ‘other’.

It is often assumed that low success rates in patients on the Category II regimen are due to the increased prevalence of drug resistance in this group, which is associated with higher rates of treatment failure and relapse.^49^,^50^ With better access to molecular DST through Xpert^®^ MTB/RIF (Cepheid, Sunnyvale, CA, USA),^51^ it is increasingly possible to identify patients with RMP resistance indicative of MDR-TB, who can be managed on second-line regimens. However, concerns remain regarding patients who have culture-negative TB for whom it is not possible to determine resistance patterns, and in settings where comprehensive DST is unavailable, so RMP resistance (but not INH-R) is diagnosed using Xpert. INH-R affects 12% of previously treated cases worldwide;^52^ however, prevalence has been estimated to vary widely between regions.^53^ INH is a crucial component of TB treatment regimens—it has greater early bactericidal activity than other drugs and has the greatest impact on clinical outcomes.^54^,^55^ As the purpose of an extended empirical drug regimen for patients with recurrent TB is to treat presumed drug resistance, the regimen should primarily be targeted at treating INH-R. Unfortunately, few studies have reported outcomes in patients with INH-R who received the Category II regimen. Evidence for the treatment of INH-R is lacking; however, the few data available indicate that the addition of SM to the intensive phase is associated with improved outcomes.^5^,^56^

The overall pattern of outcomes in this study suggested a lower rate of successful retreatment in patients who had failed initial anti-tuberculosis treatment than in patients who had relapsed following treatment. The high rate of treatment success in ‘relapsed’ patients may be largely explained by the fact that patients in this group have confirmed TB disease. Furthermore, some ‘relapses’ may in fact be reinfections with susceptible strains. In well-run TB control programmes, failure on first-line treatment is rare and more commonly associated with drug-resistant TB than other categories of retreatment.^57^ This may well account for lower success rates in this group, although drug resistance patterns were not always reported. Our findings support current treatment recommendations that, in settings where rapid DST is not routinely available, patients who have failed treatment should be started on an empirical MDR-TB regimen, unless it is known that drug resistance rates are low in that population.^3^,^58^

Particularly in settings where the prevalence of drug-resistant TB is low, several other factors may contribute to poor outcomes among patients with recurrent TB. First, some patients may not have TB at all, in which case the misdiagnosis will result in treating for TB and not the true underlying disease. While several studies have examined alternative causes of smear-negative TB in patients presenting with a first episode of TB, data exploring this issue in patients being retreated are scarce.^59^,^60^ Data from this review support such a theory, as patients classified as ‘other’ seemed to do worse than those who relapsed with a confirmed TB diagnosis. This is of particular concern as they make up the largest proportion of patients prescribed a retreatment regimen.

Second, other comorbid conditions which both predispose to TB and are associated with worse outcomes may be prevalent in this group.^61^ The phenomenon of post-tuberculous lung disease is well recognised.^62–64^ By definition, as all patients receiving the Category II regimen have had a previous episode of TB, the burden of chronic lung disease in this group is likely to be high. Those with chronic lung damage may be misclassified as having recurrent pulmonary TB or may have worse outcomes even in the event of true recurrence.

HIV-TB co-infected patients are known to have worse outcomes than HIV-negative patients after an initial episode of TB.^65^ Our review suggests that this is also the case in retreatment, but the underlying mechanism for this cannot be assumed to be the same. As people established on ART live for many years, but remain at an increased risk of TB, they are more likely to have multiple TB episodes.^66^ An increased possibility of ART failure in patients who have been on long-term ART is likely to complicate the clinical picture of recurrent TB even further.

Third, due to prolonged treatment duration and the addition of SM to the retreatment regimen, drug toxicities may be common and result in discontinuation or interruption of treatment.^67^,^68^ Finally, this longer and more demanding regimen may be associated with poor adherence and increased rates of default from treatment.

Our study had some limitations. There remains a lack of information regarding the efficacy of the Category II regimen in different subpopulations, particularly among patients who have smear-negative or extra-pulmonary disease. Studies are also lacking from key regions such as South America and Eastern Europe. It should be noted that data evaluating the correlation between drug resistance patterns and outcomes on this regimen are insufficient.

Some obvious issues must be addressed if clinical outcomes on retreatment are to be improved. The need to identify drug resistance promptly to prevent inappropriate use of retreatment regimens is now widely recognised, and further expansion of molecular DST testing in this group is required to facilitate this strategy. However, the inability to diagnose INH-R in most clinical settings and the lack of evidence about how to treat INH-R are key priorities to address. If randomised trials can establish the best treatment for INH-R, there may be an argument to use regimens against INH-R empirically for all patients with recurrent TB in settings where DST is not available. Lastly, it may be that compared with patients with a first TB episode, there is a higher burden of other comorbidities, such as HIV and chronic lung disease, in this population, which also needs to be addressed if outcomes are to improve.

People who receive the Category II retreatment regimen are a complex and heterogeneous group of patients. The regimen has no clinical trial evidence base and outcomes are poor across different regions and groups of patients. There are multiple possible explanations for these poor outcomes. The challenge of improving the management of recurrent TB will need to look beyond simply identifying those patients who have MDR-TB, and begin to incorporate appropriate approaches to the management of non-MDR-TB drug resistance, as well as address the spectrum of comorbidities from which patients on TB retreatment suffer.

## Figures and Tables

**Table A:**
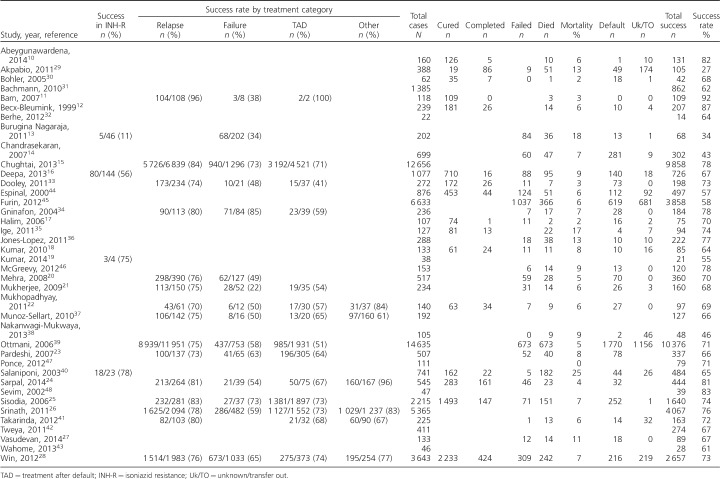
Classification of treatment outcomes for studies included in this review

## Footnotes

^*^ The appendix is available in the online version of this article, at http://www.ingentaconnect.com/content/iuatld/ijtld/2018/00000022/00000010/art00006
